# Effect of Zinc and Magnesium Compounds and Nano-Hydroxyapatite on the Physicochemical Properties and Biological Activity of Alginate and Gelatin Scaffolds for Osteochondral Defects

**DOI:** 10.3390/jfb16080300

**Published:** 2025-08-19

**Authors:** Anna Morawska-Chochół, Agnieszka Urbaś, Witold Reczyński, Ewelina Kwiecień, Magdalena Rzewuska

**Affiliations:** 1 Department of Biomaterials and Composites, Faculty of Materials Science and Ceramics, AGH University of Krakow, 30-059 Krakow, Poland; 2 Faculty of Electrical Engineering, Automatics, Computer Science and Biomedical Engineering, AGH University of Krakow, 30-059 Krakow, Poland; agnieszka.urbas98@gmail.com; 3 Department of Analytical Chemistry and Biochemistry, Faculty of Materials Science and Ceramics, AGH University of Krakow, 30-059 Krakow, Poland; wreczyn@agh.edu.pl; 4 Department of Preclinical Sciences, Institute of Veterinary Medicine, Warsaw University of Life Sciences—SGGW, 02-786 Warsaw, Poland; ewelina_kwiecien1@sggw.edu.pl (E.K.); magdalena_rzewuska@sggw.edu.pl (M.R.)

**Keywords:** alginate, gelatin, antibacterial, zinc, magnesium, hydroxyapatite, scaffolds

## Abstract

Composite scaffolds based on a hydrogel matrix modified with hydroxyapatite, magnesium, or zinc compounds are promising for filling and regenerating osteochondral defects due to the specific biological properties of these modifiers. The aim of this work was to evaluate the influence of hydroxyapatite, nano-hydroxyapatite, magnesium chloride, and zinc oxide on mechanical properties, swelling ability, behavior in a simulated biological environment (ion release, stability, bioactivity), and antibacterial effects. Furthermore, the influence of the hydrogel matrix (alginate, gelatin, alginate/gelatin) on the selected properties was also assessed. The results showed that the addition of ZnO improved the mechanical properties of all types of matrices most effectively. Additionally, zinc ions were gradually released into the environment and partially incorporated into the formed apatite. The released zinc ions increased the inhibition zones of *Staphylococcus aureus* growth; however, this effect was observed only in scaffolds with an alginate matrix. This indicates that hydrogel plays a key role in antibacterial effects, beyond the contribution of antibacterial additives. No effect of magnesium on bacterial growth inhibition was observed despite its rapid release. Magnesium ions promoted efficient secretion of apatite during incubation, although it was not stable. The addition of nano-HAP significantly increased the stability of the apatite precipitates.

## 1. Introduction

Treatment of cartilage defects, including osteochondral defects, remains a current challenge due to the specificity of cartilage tissue, the lack of metabolically active cells, the lack of vascularization, and innervation [1,2]. Currently, several approaches are used in the treatment of cartilage, the most promising of which seems to be the approach involving filling the defect with a gradually degradable scaffold that will stimulate tissue regeneration in a controlled manner and will provide an appropriate place for cell migration and proliferation [1,2]. Hydrogels are a group of materials that offer such possibilities thanks to their specific properties, such as a similarity to the natural extracellular matrix (a high content of water thanks to a high degree of swelling) and the possibility of modification with a biologically active substance, the release of which can be controlled. Alginate and gelatine are common hydrogels that belong to polysaccharides and peptides, respectively [2,3,4,5].

Hydroxyapatite (HAP), as a natural bone component, is known for its bioactive and osteoconductive properties. Moreover, as a ceramic additive, it allows for a controlled modification of the mechanical parameters of composite scaffolds with an organic matrix by selecting its content and particle size [6,7]. In the newest literature, Zhao et al. described the potential of nanohydroxyapatite used in a bilayer scaffold for subchondral bone rehabilitation [8]. The scaffold consisted of gelatine, carboxymethyl chitosan, and oxidative sodium alginate as the matrix of the biomaterial.

Magnesium compounds exhibit bioactive properties and stimulate bone formation [9]. Magnesium, as a bioelement, is essential for the proper functioning of the body, and magnesium substitutions also occur in biological apatite. In addition, it plays a role in the proliferation of mesenchymal stem cells and chondrogenesis [10,11,12] and has antibacterial effects [13]. Xie et al. demonstrated the excellent antibacterial effect of magnesium ions against methicillin-resistant *Staphylococcus aureus* (MRSA) and *Escherichia coli* [13]. Alarcón et al. noticed the connection between MgCl_2_ antimicrobial activity and the pH of the medium [14]. This effect was observed at pH values lower than 5 in the presence of anionic bases [14].

ZnO is known for its antibacterial properties and is a common modifier of biomaterials [15,16]. Mousa et al. used ZnO nanoparticles (5 and 10 wt.%) to modify the polylactide layer coated on magnesium alloys for bone tissue engineering applications [15]. Shitole et al. described the positive role of ZnO and HAP in the modification of electrospun polycaprolactone nanofibers, which had antibacterial activity against *Escherichia coli* and *Staphylococcus aureus*, proper mechanical stability, and good cell viability and proliferation for scaffolds with a ZnO concentration range of ≤10% [16]. However, there are also papers describing the toxic effect of zinc oxide correlated with particle size and its concentration [17,18]. Nair et al. showed that toxicity is related to nanoparticles [17]. Bashir et al. studied the level of ZnO nanoparticle toxicity in different organs in relation to dose rates in a mouse model. They described that for a dose of 0.14 mg/kg, no toxic effect was observed [18].

Composite scaffolds based on a hydrogel matrix modified by hydroxyapatite, as well as magnesium and zinc compounds, are promising in their application for filling and regenerating osteochondral defects. The use of proposed modifiers enables targeted modification of the physicochemical and biological properties of hydrogels. The novelty of this work is the multifunctionality of scaffolds created by connecting hydrogels with different modifiers with bioactive, regenerative, and antibacterial properties. Moreover, this approach should enable multifunctional action by ensuring the necessary mechanical and microstructural parameters and gradual degradation, as well as initiating and stimulating regenerative processes in a comprehensive way. The aim of this work was to estimate the influence of hydroxyapatite, nano-hydroxyapatite, magnesium chloride, and zinc oxide on mechanical properties, swelling ability, behavior in simulated biological environment (ion release, stability, bioactivity), and antibacterial effects. Moreover, the influence of a hydrogel matrix (alginate, gelatin, alginate/gelatin) on the selected properties was assessed. As has been shown, the effect of modifiers can be amplified or inhibited depending on the hydrogel type.

## 2. Materials and Methods

### 2.1. Materials

The following reagents were used in the fabrication of scaffolds: gelatin (CAS 9000-70-8, POCH S.A., Gliwice, Poland; Mw = 15,000–400,000 Da); alginic acid (CAS 9005-38-3, Acros Organics, Geel, Belgium; Mw = 450,000–550,000 Da), hydroxyapatite powder, HAp (CAS 1306-06-5, Acros Organics, Geel, Belgium; particles size 7.10 µm ± 1.15 µm); hydroxyapatite nanopowder, nHAp (size 99% < 100 nm; average particle size 20–80 nm; specific surface area 15–50 m^2^/g; CAS 1306-06-5, n-Gimat, Lexington, Kentucky, USA); magnesium chloride hexahydrate, MgCl_2_∙6H_2_O (CAS 7791-18-6, POCH S.A., Gliwice, Poland); zinc oxide, ZnO (CAS 1314-13-2, Macron Fine Chemicals, Center Valley, Pennsylvania); calcium chloride, CaCl_2_ (CAS 10043-52-4, POCH S.A., Gliwice, Poland); N-(3-Dimethylaminopropyl)-N′-ethylcarbodiimide hydrochloride, EDAC (CAS 25952-53-8, Sigma-Aldrich, St. Louis, MO, USA); and Phosphate-Buffered Saline, PBS (Sigma-Aldrich, St. Louis, MO, USA).

### 2.2. Scaffolds Fabrication

Three groups of composite scaffolds with different hydrogel matrices were obtained: alginate (1), gelatin (2), and alginate/gelatin 1:4 (3) matrix. In every group, 5 different compositions of additives were used in the same way. The type and share of modifiers, together with scaffold code, are presented in Table 1.

**Figure 1:**
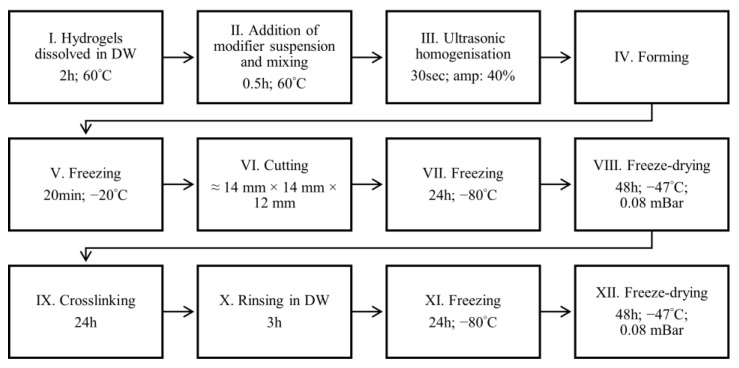
Stages of scaffold manufacturing.

**Figure 2:**
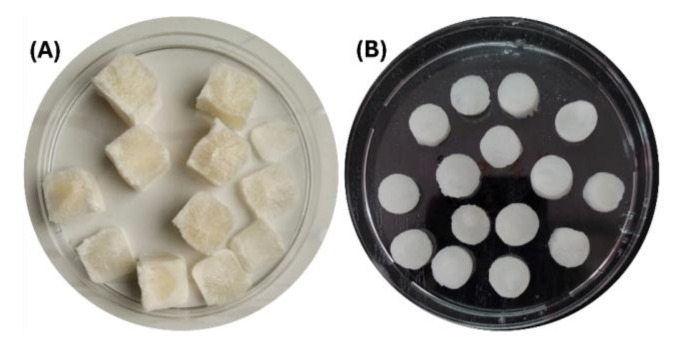
Example of scaffolds used in the studies of physicochemical properties (**A**) and antibacterial activity (**B**).

The stages of scaffold manufacturing are presented in Figure 1. First, three hydrogel solutions were prepared by dissolving hydrogel powders in distilled water (DW): sodium alginate (2.25 g/25 mL), gelatin (2.25 g/25 mL), and sodium alginate together with gelatin (0.4 g and 1.6 g, respectively, in 25 mL). This step was repeated 5 times to obtain 5 of the same solutions in every group. The appropriate modifiers were suspended in 5 mL of DW in 5 compositions presented in Table 1 (No 1–5) and then combined with 5 hydrogel solutions in every 3 groups of hydrogels. The additives were used in the following weights: 127.66 mg of HAp (No 1–5); 83.33 mg of MgCl_2_∙6H_2_O (No 2); 127.66 mg of MgCl_2_∙6H_2_O (No 3); 0.20 mg of nanoHAp (No 4); and 83.33 mg of ZnO (No 5). The weight percentages of MgCl_2_∙6H_2_O were 4 wt.% or 6 wt.%, which correspond, respectively, to 1.90 wt.% and 2.88 wt.% of MgCl_2_ (0.49 wt.% and 0.74 wt.% of Mg related to the dry mass of polymer). The weight percentage of ZnO was 4 wt.% (the content of Zn was 3.34 wt.% related to the dry mass of polymer). The solutions with additives were ultrasonicated. Next, the solutions were cast into a Petri dish and freeze-dried for antibacterial test (stages V and VI in Figure 1 were skipped) or cast to a rectangular mold (12 cm × 14 mm × 14 mm), frozen, cut, and freeze-dried for the other tests (stages V to VIII in Figure 1). The freeze-drying process was performed using LABCONCO freeze-dryer (Kansas City, Missouri, USA). Next, the samples were crosslinked in different ways depending on the hydrogel type: with an alginate matrix in 0.5 wt.% CaCl_2_ (in DW), with gelatin matrix in 1 wt.% EDAC (in DW), or with alginate/gelatin matrix in the solution of EDAC and CaCl_2_ (1 wt.%, and 0.5 wt.% respectively in DW). Next, steps X to XII were performed (Figure 1). Finally, the dry samples for antibacterial tests were cut into the shape of a disc with a 10 mm diameter. The scaffolds obtained are presented in Figure 2.

### 2.3. Characterization

#### 2.3.1. The Initial Water Content, PBS Absorption Capacity (Swelling Ability), and Mass Changes During Incubation in PBS

Wet samples were weighed (W_w_) just after fabrication (after step X in Figure 1) (*n* = 3). Next, the scaffolds were freeze-dried following steps XI and XII in Figure 1. The dry scaffolds after measuring the weights (W_d_) were incubated in PBS for 4 weeks (pH = 7.4; 37 °C). Only scaffolds with an alginate matrix were incubated. Scaffolds were weighed (W’_w_) after 2 and 4 weeks (the excess PBS was removed from the sample’s surface with filter paper), freeze-dried (−80 °C for 24 h, next lyophilized for 48 h), and weighed again (W’_d_).

The initial water content (S) was calculated according to Equation (1):

S = (W_w_ − W_d_)/W_d_ × 100%, (1)

where W_w_ and W_d_ represent wet weight directly after fabrication (before drying) and dry weight after freeze-drying, respectively.

The PBS absorption capacity during incubation (S’) was calculated according to Equation (2):

S’ = (W’_w_ − W’_d_)/W’_d_ × 100%, (2)

where W’_w_ and W’_d_ represent wet weight and dry weight after incubation, respectively.

Mass changes during incubation (W) were calculated for dry samples according to Equation (3):

W = (W’_d_ − W_d_)/W_d_ × 100%, (3)

where W_d_ and W’_d_ represent dry weight before and after incubation in PBS, respectively.

#### 2.3.2. Zinc and Magnesium Release

Release of zinc and magnesium ions from scaffolds in PBS (pH = 7.4) solution was measured during 2 weeks of incubation at 37 °C by collecting 1 mL of supernatants after 4, 7, and 14 days of sample incubation. The collected volume of supernatants was replaced with fresh PBS. The ion concentration was determined by Atomic Absorption Spectrometry (AAS) in flame technology using a Model 3110 spectrometer (Perkin-Elmer Corp., Killeen, TX, USA).

#### 2.3.3. Antibacterial Activity Tests

The following scaffolds with alginate and alginate/gelatin matrices were tested: ALG_6H and ALG_GEL_6H as controls, and ALG_6H_6Mg, ALG_GEL_6H_6Mg, ALG_6H_4Zn, and ALG_GEL_6H_4Zn. The scaffolds for antibacterial tests were selected in such a way that the influence of the matrix, as well as zinc and magnesium, could be assessed. Dry disc-shaped samples (Figure 2B) had a diameter of 10 mm and a height of 1 mm. Scaffolds were moistened with sterile distilled water and swollen to approximately 14 mm × 2 mm (diameter and height, respectively). However, the final size was determined by the swelling ability of the matrices. The study of antibacterial activity was conducted against the following strains: *Escherichia coli*—ATCC 25922 (*E. coli*); *Pseudomonas aeruginosa*—ATCC 27853 (*P. aeruginosa*); and *Staphylococcus aureus*—ATCC 25923 (*S. aureus*). Bacterial suspensions were prepared in PBS (potassium chloride 2.7 mM, potassium phosphate monobasic 1.76 mM, sodium chloride 0.137 M, sodium phosphate dibasic 10.1 mM, pH 7.4; Thermo Fisher Scientific, Waltham, MA, USA) using 24 h cultures of bacteria on Columbia blood agar (Graso Biotech, Starogard Gdański, Poland) and adjusted to a density of 0.5 of the MacFarland standard. The samples were placed into sterile Petri plates (one sample per plate) and Mueller–Hinton agar (bioMérieux, Lyon, France) at 45 °C, containing a specific bacterial strain (0.1 mL of a bacterial suspension per 20 mL of the medium), was poured over them. After medium solidification, the plates were incubated at 37 °C, +/−0.1 °C, under aerobic conditions. The antibacterial activity of the hydrogels tested was evaluated by measuring the growth inhibition zone around the sample after 24 and 48 h of incubation. In the case of a visible inhibition zone, the distance between the edge of the sample and the distinct edge of the bacterial growth zone was measured. The test was performed in triplicate for each sample/bacteria system.

#### 2.3.4. Mechanical Tests

The mechanical properties of the hydrogel scaffolds were investigated using the Zwick 1435 universal testing machine (ZwickRoell, Ulm, Germany). A static uniaxial compression test was carried out with the following parameters: initial force: 0.1 N; compression modulus speed 1 mm/min; and test speed: 5 mm/min. The rectangular-shaped specimens with dimensions of 14 × 14 × 12 mm (width × depth × height) were evaluated in the dry state. The compression modulus (Ec) and the compressive stress at 50% relative contraction (σ_ε=50_) were determined. The mechanical parameters were also evaluated for scaffolds with an alginate matrix after 2 and 4 weeks of incubation in PBS (pH = 7.4, 37 °C). Samples were freeze-dried (−80 °C for 24 h) and lyophilized for 48 h before mechanical test.

#### 2.3.5. Microstructural Observations with Elemental Analysis (SEM/EDS)

The morphology of the samples and their elemental composition were examined using Scanning Electron Microscopy SEM (Nova Nano SEM 200, FEI Europe Company, Eindhoven, The Netherlands) with Energy Dispersive Spectrometry EDS (EDAX Company, Mahwah, NJ, USA) at the Department of Ceramics and Refractories, Faculty of Materials Science and Ceramics at the AGH University of Krakow. Prior to testing, the samples were sprayed with carbon. This study was performed for initial samples (scaffolds with alginate and alginate/gelatin matrix) and after 2 and 4 weeks of incubation at 37 °C in PBS solution (scaffolds with alginate matrix).

#### 2.3.6. Infrared Structural Analysis (FTIR-ATR)

Infrared structural analysis was performed using a Bruker Tensor 27 spectrometer with TGA-IR equipment on the germanium crystal (Bruker Corporation, Billerica, MA, USA). The spectra were obtained at room temperature; 32 scans were performed for each sample with a resolution of 4 cm^−1^.

#### 2.3.7. Size Distribution of the ZnO Particles

The primary size distribution of the dry ZnO powder was measured using Malvern Mastersizer 3000e (Malvern Panalytical, Malvern, Worcestershire, UK). The samples were administered to the laser cell using a venturi nozzle. The particles were dispersed at 4 bars with the feed rate adjusted to each sample to obtain sufficient laser obscuration. The background was measured for 10 s while the single sample measurement was carried out within 3 s. Altogether, 11 measurements were carried out. The data was analyzed with dedicated Mastersizer v3.88 software (Malvern Instruments Ltd., Malvern, Worcestershire, UK) using the Mie particle model. The D10, D50, and D90 (D—diameter in micrometers) and span values were derived from the resulting volumetric distribution curve, in which D50 represents the median value; i.e., 50% of the total volume of particles had a diameter smaller than the D50 and 50% had a diameter larger than the D50, according to the methodology described in more detail by Kwiecień et al. [19].

#### 2.3.8. Statistical Analysis

All data were obtained from three parallel samples in every group of materials. Data were calculated using the mean ± standard deviation.

## 3. Results and Discussion

### 3.1. Mechanical Properties

**Figure 3:**
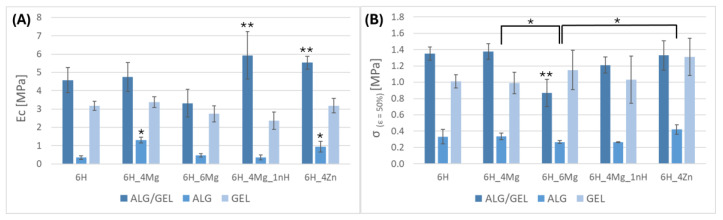
Compression modulus Ec (**A**) and compression stress σ (ε = 50%) (**B**) of composite scaffolds with various matrices. Statistically significant differences at *p* < 0.01; * ALG, ** ALG/GEL.

The results of the mechanical studies of composite scaffolds with three types of matrices, ALG/GEL, ALG, and GEL, are presented in Figure 3. The lowest mechanical parameters were obtained for the composites with an ALG matrix, for which the highest value of compression stress σ_(ε=50%)_ equal to 0.42 MPa was recorded for the scaffold containing zinc (ALG/6H_4Zn). Mechanical parameters which were about three times greater were obtained for the analogous composites with a GEL matrix. In this case, the highest stress value of 1.31 MPa was also found in the zinc composite (GEL_6H_4Zn). The significant results are those obtained for the ALG/GEL composites, which indicate that the combination of gelatin and alginate significantly increased the value of the compression modulus and compression stress compared to hydrogels with alginate or gelatin alone. This effect is particularly evident in the case of σ_(ε=50%)_ of the ALG/GEL_6H and ALG/GEL_6H_4Mg scaffolds (the differences for the other materials are not statistically significant) and in the case of the compression modulus of all materials except ALG/GEL_6H_6Mg, for which a similar trend is maintained, but the values are within the limits of statistical error. The highest value of stress of approx. 1.35 MPa was observed for the scaffolds modified by 6H, 6H4Mg, and 6H_4Zn. The observed relationship indicates a synergistic effect, where the simultaneous use of alginate and gelatin allows the creation of the structure of an interpenetrating polymer network (IPN), and in consequence, a strengthening effect is obtained [20,21,22]. IPN is connected with molecular crosslinking and chain entanglement, combined with the features of physical and chemical crosslinking. Therefore, multifunctional, more complex structures and better mechanical properties can be achieved [23].

Comparing the influence of additives on the mechanical parameters of scaffolds, the effect of zinc on the increase in value of σ_(ε=50%)_ is significantly visible, especially with the ALG and GEL matrices. The reinforcement mechanism of the scaffolds modified with micrometric ZnO (D50 = 24.2 ± 8.7 μm; Span = 28.6 ± 11.6; particle size > 100 nm) was typical for composites in which the reinforcing phase was a modifier in the form of ceramic particles. The strengthening effect was due to the reduction of the mobility of polymer chains and the stiffening of the structure. For the composites with an ALG/GEL matrix, a lower weight percentage of MgCl_2_ had a more positive effect on the mechanical properties. The effect of nano-HAp varies greatly depending on the matrix type. Moreover, a high dispersion of results is also visible for the scaffolds with a gelatin matrix, suggesting a heterogeneous distribution of modifiers, which is particularly difficult due to the high viscosity of the solution and the presence of other additives. This results in the absence of a reinforcing effect of the nano-modifier.

### 3.2. The Initial Water Content in Hydrogel Scaffolds

**Figure 4:**
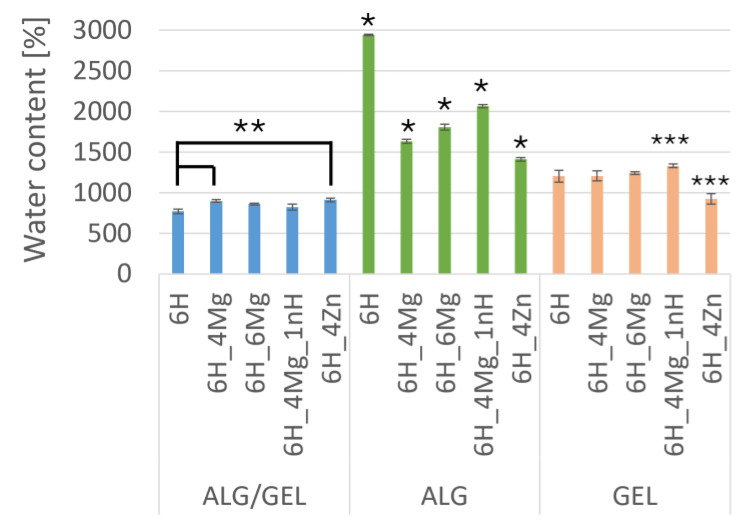
The initial water content of hydrogel scaffolds. Statistically significant differences at *p* < 0.01; * ALG, ** ALG/GEL, *** GEL.

The initial water content in the gelatin scaffolds was significantly lower compared to the alginate ones (Figure 4). This is the effect of the different crosslinking mechanisms of gelatin and alginate. EDC caused chemical crosslinking of gelatin with amide groups (–C(=O)–NH–), which were created by carboxylic groups and the free amine groups [24,25]. Alginate was crosslinked by a weaker ionic bond according to the “egg-box” model; therefore, its molecular structure was less compact and its ability to absorb water was higher [26]. The lowest value of water content in the scaffolds with alginate/gelatin matrix indicates the synergic effect connected with the interpenetrating polymer network [23], which decreased the flexibility of the scaffolds, confirmed by the highest compression modulus (Figure 3A).

For scaffolds with an alginate matrix, a significant impact of additives on the decrease in water content can be observed. It can be especially visible in the case of ALG_6H_4Zn and ALG_6H_4Mg and correlates with the increase in scaffold stiffness, confirmed by the highest compression modulus of this scaffold in the group with an alginate matrix. It can be related to the creation of additional ionic bonds in the presence of Mg^2+^ ions and the stiffening of the molecular structure. A higher water content for ALG_6H_6Mg can be connected with the higher amount of water introduced in the MgCl_2_ hydrate and, in consequence, the higher porosity created during the freeze-drying process [27], whereas the higher water content for ALG_6H_4Mg_1nH indicates the role of nanoparticles in loosening the arrangement of hydrogel molecules. For the scaffolds with ZnO, the mechanism is probably different; ceramic particles may hinder the movement of alginate molecules.

The swelling ability correlated with the measured water content of a scaffold plays a critical role in determining its feasibility for practical biomedical applications, especially active substance delivery and tissue engineering [28]. A scaffold with good swelling ability can absorb bodily fluids and allow the diffusion of nutrients and oxygen to cells. This is essential for cell survival, proliferation, and tissue regeneration. Moreover, swelling enables controlled release of therapeutic agents embedded in the scaffold. Excessive swelling can compromise mechanical integrity or cause the scaffold to lose its shape. Optimal swelling should strike a balance between fluid uptake and structural stability. The obtained parameters correlated with the next result showed that the water content of alginate with MgCl^2^ and ZnO modifiers is proper for application.

### 3.3. Release of Mg^2+^ and Zn^2+^ Ion from Scaffolds

**Figure 5:**
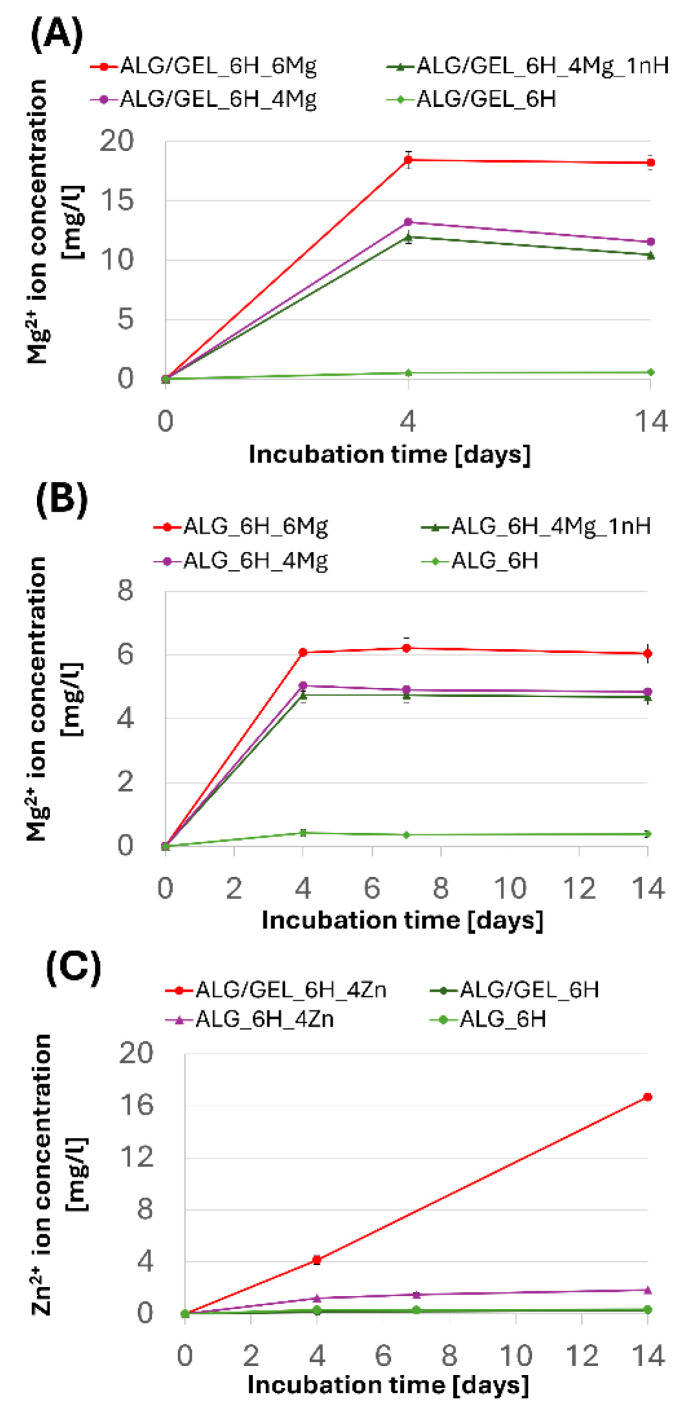
Changes in ion concentration in supernatants during 2 weeks of incubation in PBS: release of Mg^2+^ from ALG/GEL (**A**) and ALG scaffolds (**B**); release of Zn^2+^ from ALG/GEL and ALG scaffolds (**C**).

**Figure 6:**
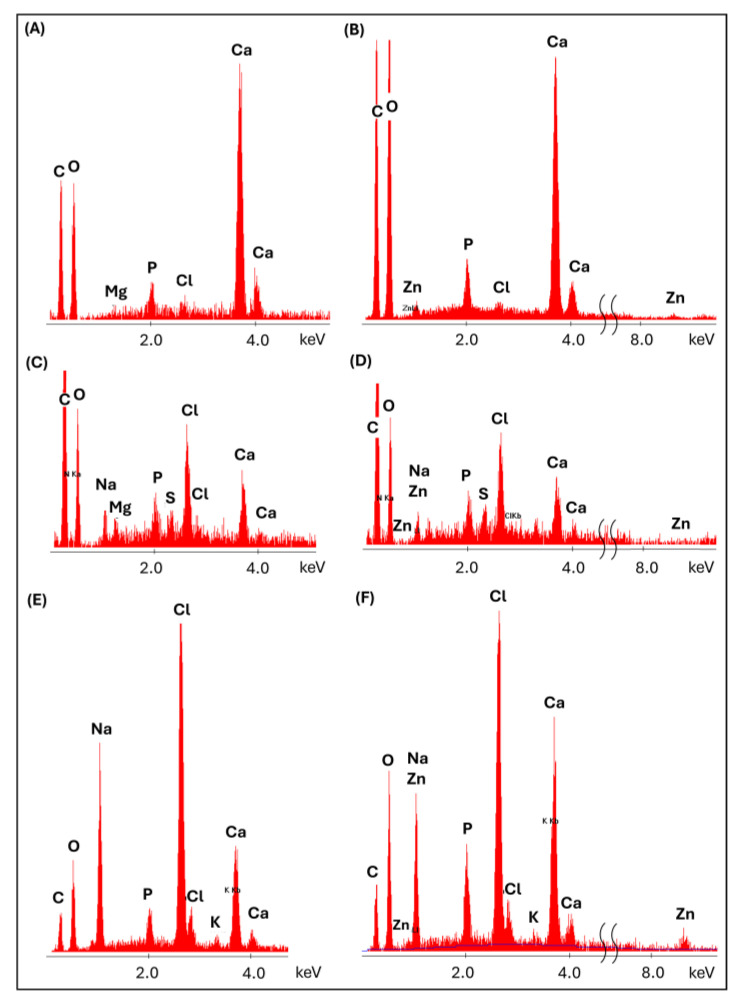
Average EDS analysis of initial scaffolds: ALG_6H_6Mg (**A**), ALG_6H_4Zn (**B**), ALG/GEL_6H_6Mg (**C**), and ALG/GEL_6H_4Zn (**D**), and after 2 weeks incubation in PBS, ALG_6H_6Mg (**E**), and ALG_6H_4Zn (**F**).

Mg^2+^ and Zn^2+^ ion release was assessed during 2 weeks of incubation of the scaffolds in PBS. The release of ions from the alginate and alginate/gelatin scaffolds was measured (Figure 5). Composites with a gelatin matrix did not show sufficient stability during incubation in PBS and were excluded from further studies. The maximum concentration of released Mg^2+^ ions was measured after the first few days (the first measurement period) for both types of matrices (ALG/GEL and ALG) (Figure 5A,B). Importantly, the maximum amount of released magnesium was only part of the total amount of this modifier incorporated into the scaffolds during their formation. For the alginate matrix, it was 4.9 mg/L (ALG_6H_4Mg) and 6.2 mg/L (ALG_6H_6Mg), which accounted for approx. 11% of the total amount of magnesium ions initially incorporated into the samples. The amounts of magnesium ions released from the alginate/gelatin matrix were significantly higher than from the alginate matrix with analogous additives, but it was still about 30% of the total amount of magnesium used during forming (the measured concentration was 11.9 mg/L for ALG/GEL_6H_4Mg and 18.4 mg/L for ALG/GEL_6H_6Mg). EDS analysis showed the lack of magnesium in scaffolds after 2 weeks of incubation (Figure 6E), which confirmed that the total amount of incorporated magnesium ions was released during this time. It indicates that most of the magnesium was released during the cross-linking process. EDS analysis of scaffolds before incubation showed that the amount of magnesium remaining in the scaffolds after cross-linking was significantly higher in the ALG/GEL matrix compared to ALG (Figure 6A,C), probably because of the chemical reactions of the hydrogel with EDC. This explains the higher amount of magnesium released from the ALG/GEL scaffolds. The concentrations of released Mg^2+^ ions from every type of scaffold were within the safe norm for the body, which, according to the literature, is 26.5 mg/L. This means that these scaffolds could contribute to the promotion of the formation of new bone, without the local toxic concentration of Mg^2+^ ions.

Zn^2+^ ions were released more slowly, more gradually and in much smaller quantities than magnesium ions, which was particularly visible for the ALG/GEL_6H_4Zn sample (Figure 5C). The concentration recorded after 14 days was 16.7 mg/L for the ALG/GEL matrix, which was only 13.6% of the total amount of zinc ions contained in the sample. The maximum concentration of zinc ions released from the ALG matrix was only 1.85 mg/L, which is 0.7% of the total amount of zinc in this material. This indicates that a much larger amount of zinc ions was released from the ALG/GEL matrix than from the ALG matrix, confirming the significant role of the matrix in the release process. The faster release of ions from alginate/gelatin compared to the alginate matrix was a consequence of the lower stability of alginate/gelatin in PBS. In this case, the release rate was not correlated with the swelling ability. Water in the hydrogel is a transport medium enabling the diffusion of substances, and the degree of cross-linking of the polymer matrix influences the properties of their transport in the material [29]. The faster degradation of alginate/gelatin scaffolds is related to the destruction of bonds and, in consequence, allows for easier migration of ions.

The concentration of released Zn^2+^ ions was significantly lower than that of Mg^2+^ ions. Differences in the release of Mg^2+^ and Zn^2+^ ions were related to the different solubility of MgCl_2_ and ZnO, and therefore quite different mechanisms. Magnesium chloride is soluble in an aqueous medium, easily providing magnesium ions. In contrast, the release of zinc ions from ZnO occurs through chemical reactions in an aqueous medium rather than by simple dissolution, as ZnO is practically insoluble in an aqueous medium. In future studies, the use of poorly soluble magnesium salts (MgO; MgCO_3_) and highly soluble zinc salts (ZnCl_2_; ZnSO_4_) is planned to validate the conclusions. The slower release of Zn^2+^ ions can also be connected to their incorporation into the structure of alginate as an ionic bond, thus strengthening the materials during incubation, which was visible in the mechanical studies (in Section 3.5.2). EDS analysis after 2 weeks of incubation confirmed the zinc incorporation into the alginate structure (Figure 6F) but also into the apatite precipitation (described in Section 3.5.3).

### 3.4. Antibacterial Activity

In this study, an antibacterial effect was noted only against *Staphylococcus aureus*. In the study using *Escherichia coli*, zones of less intense growth around the scaffolds were observed but were not so significant that a clear inhibitory effect could be confirmed. No growth-inhibitory effect was observed for *Pseudomonas aeruginosa*. The measurements of the growth inhibition zones for the *Staphylococcus aureus* strain after 24 h incubation are collected in Table 2. The dimension of the inhibition zones did not change after the incubation was prolonged to 48 h.

**Figure 7:**
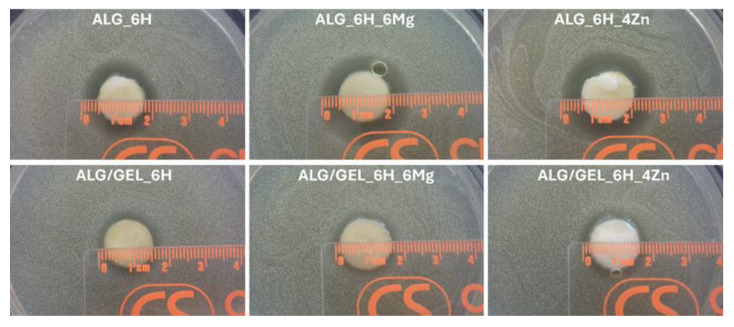
Inhibition zones of *S. aureus* growth after 24 h of incubation.

The differences in the release of Mg^2+^ and Zn^2+^ ions from two types of matrices, ALG and ALG/GEL, did not correlate with the observed differences in inhibition of bacterial growth (Figure 7). In the case of the ALG matrix samples, significantly larger growth inhibition zones were observed, even for the control sample, only with the addition of HAP (ALG_6H). This phenomenon can be explained by the effect of divalent metal ions on antibacterial activity described by Luque-Agudo et al. [30]. The authors showed that the positive charge of Ca^2+^ cations promoted bacterial adhesion, which facilitates the action of ions on bacteria. Electrostatic interactions were the cause of the attraction between the negatively charged bacterial cell wall and alginate surfaces doped with the Ca^2+^ [30,31]. de Kerchove and Elimelech [32] confirmed the effect of divalent cations on the adhesion of *P. aeruginosa* on alginate films, which was related to structural changes in the alginate caused by the presence of these cations. The authors concluded that under physiological conditions, unspecific electrical forces were one of the main factors modulating the initial approach of bacterial cells to alginate through divalent cation bridges.

The largest zone of bacterial growth inhibition was observed for the scaffold with zinc (ALG_6H_4Zn), indicating its pronounced antibacterial activity against the *Staphylococcus aureus* strain, but only in the case of the ALG matrix. No inhibitory effect of zinc on bacterial growth was observed for an analogous sample with an ALG/GEL matrix (ALG/GEL_6H_4Zn), despite a higher concentration of zinc released from this scaffold. This indicates that gelatin was blocking the antibacterial effect, possibly due to different environmental conditions. The antibacterial effect of the scaffolds with zinc may result not only from the release of zinc ions but also from the properties of ZnO itself. ZnO particles exhibit a positive charge in the physiological environment that may be responsible for the stronger electrostatic interaction with the negatively charged bacterial membrane. This property of ZnO facilitates adhesion to the cell surface and disruption of the membrane structure [33]. Due to the small size of the ZnO particles (D50 = 24.2 ± 8.7 μm; Span = 28.6 ± 11.6; particle size > 100 nm) and the swelling of the hydrogel, it was possible for the smallest particles to diffuse into the solution and facilitate contact with bacteria.

Importantly, in both types of matrices, no effect of Mg^2+^ ions on the pathogen growth was observed, despite their rapid release. In other publications, the antibacterial effect of magnesium against methicillin-resistant *S. aureus* (MRSA) and *Escherichia coli* has been observed [13,28,34]; however, the authors observed the relationship between the type of magnesium compounds and antibacterial activity. Poly(lactide-co-glycolide) microspheres containing MgO and MgCO_3_ (in a 1:1 wt ratio) displayed inferior antibacterial activity compared to that with MgO [28]. The literature showed the antibacterial activity of ZnO against *E. coli* and *S. aureus* [16] as well as the inhibition of biofilm formation by *P. aeruginosa* [35]. Concluding the results obtained in the presented studies, it is also possible that the magnesium and zinc activity was neutralized by the alginate/gelatin matrix, e.g., by changing the ambient parameters of the samples. According to the literature data, the antibacterial activity of a given compound is possible when appropriate environmental conditions, such as appropriate pH, temperature, chemical composition, etc., are provided at the same time [14]. Acidification of the environment is not conducive to the growth of bacteria, while magnesium compounds tend to alkalize the environment [34]. The literature indicates the complexity of the problem and the multi-parameter dependence; therefore, this requires further research.

### 3.5. Behavior of Scaffolds in a Simulated Biological Environment

Tests were performed for the group of scaffolds with the alginate matrix because of their highest antibacterial activity and greatest stability in PBS.

#### 3.5.1. Changes in Scaffold Weight After Incubation in PBS

**Figure 8:**
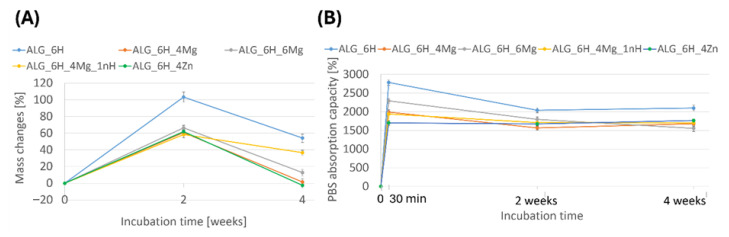
Mass changes (**A**) and absorption capacity (**B**) for alginate scaffolds during 4 weeks of incubation in PBS.

The alginate scaffolds showed high stability during 4 weeks of incubation in PBS, regardless of the modifiers used (Figure 8A). None of the samples showed a significant loss in weight determined for dry samples (after freeze-drying). It is noteworthy that after 2 weeks of incubation, the samples showed a significant increase in weight, which exceeded 100% for ALG_6H, while for the other samples it reached about 60%. This phenomenon indicates that a significant amount of ions from the PBS solution was absorbed during the swelling process and remained in the alginate structure during the drying process. EDS analysis (Figure 6E,F) showed that these were mainly potassium ions and sodium chloride. Moreover, on the surface of the scaffolds, calcium phosphate and sodium chloride precipitates occurred, which were confirmed in FTIR and SEM/EDS studies (Section 3.5.3). Weight growth of scaffolds may also be associated with the chemical incorporation of water during incubation by the chemical binding of OH-groups with the hydrogel structure. The highest weight of ALG_6H was connected with its highest absorption capacity (Figure 8B) and swelling ability described in Figure 4. The weight growth after 4 weeks of incubation was lower than after 2 weeks, and clear differences between the individual materials appeared. This indicates that ion migration occurred bidirectionally, and re-dissolution of precipitates could also take place. It is also likely that low-molecular-weight alginate molecules were removed from scaffolds. Weighing wet samples soaked in PBS solution allowed the assessment of the absorption capacity of scaffolds (Figure 8B). These results showed the highest absorption at the beginning of incubation (30 min). This parameter decreased after 2 weeks in PBS for all samples except ALG_6H_4Zn and then remained stable. Moreover, ALG_6H_4Zn showed the lowest absorption capacity after 30 min, which correlates with the swelling capacity and the lowest elasticity (described in Figure 3A and Figure 4).

#### 3.5.2. Changes of the Mechanical Parameters of Scaffolds After Incubation in PBS

**Figure 9:**
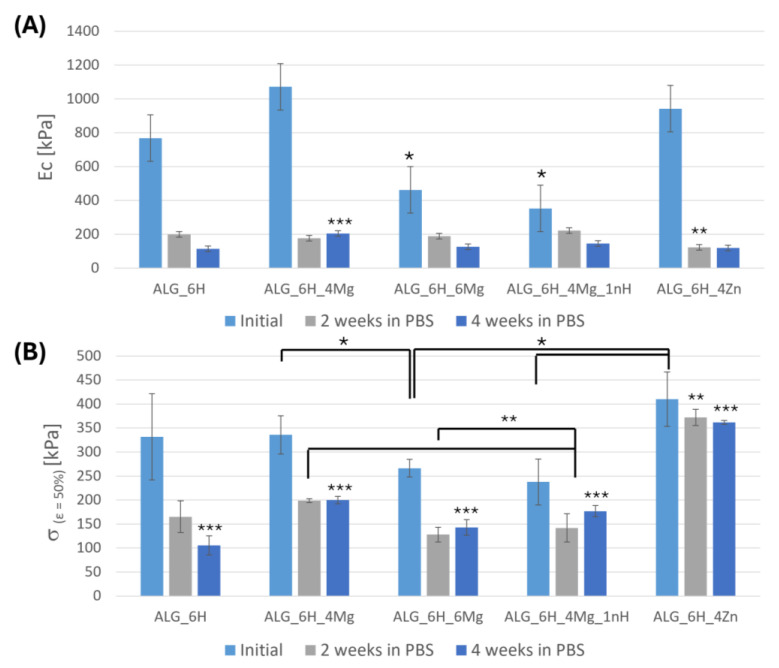
Compression modulus (Ec) (**A**) and compression stress (σ_(ε=50%)_) (**B**) of composite scaffolds with alginate matrix after 2 and 4 weeks of incubation in PBS. Statistically significant differences at *p* < 0.01; * initial, ** 2 weeks in PBS, *** 4 weeks in PBS.

The degradation rate of the scaffolds was also estimated on the basis of the mechanical property changes after incubation in PBS (Figure 9). A decrease in compression modulus and strength was observed with incubation time in PBS for all scaffolds; however, the intensity of these changes depended strictly on the type of modifier. The highest decreases in the value of the compression modulus, more than 80% of the initial modulus after 2 weeks of incubation, were observed for samples with the addition of zinc oxide (ALG_6H_4Zn) and a lower amount of magnesium chloride (ALG_6H_4Mg). Similar values were noted after 4 weeks of incubation. For the rest of the scaffolds, changes in compression modulus became more gradual. Changes in the compression modulus were probably related to the release of these modifiers that originally significantly increased the stiffness of the hydrogel. After incubation, the differences between particular materials were not so noticeable. Moreover, an increase in the swelling ability (Figure 4) and the loosening of the molecular arrangement of the hydrogel correlated with the higher pore size might cause a decrease in compression modulus.

The differences in σ_(ε=50%)_ in most samples are within the measurement error range and amount to about 40–52% after 2 weeks of incubation. For the ALG_6H_4Mg and ALG_6H_6Mg samples, no significant difference in the value of this parameter was observed between 2 and 4 weeks after incubation, indicating that the processes that affected this parameter occurred up to week 2 and are probably related to the release of magnesium ions. For the reference sample (ALG_6H), the strength loss increased markedly after 4 weeks and reached the highest value among the tested materials of 68%. This indicates that the modifiers used in the form of magnesium, zinc compounds, and nano-HAp significantly increase the hydrolytic stability of the scaffolds. Unlike other materials, an increase in the value of σ_(ε=50%)_ was observed for ALG_6H_4Mg_1nH after 4 weeks compared to the result obtained after 2 weeks. This may be the result of a large statistical error after 2 weeks of incubation, related to the heterogeneity of these samples.

Compression stress was the most stable during incubation for ALG_6H_4Zn, for which the reduction σ_(ε=50%)_ was only 9.27% after 2 weeks and 11.78% after 4 weeks of incubation, although the highest loss of the compressive modulus was observed for this material. This can be related to the creation of stronger molecular bonds in the presence of Zn^2+^ ions, which, besides releasing them to the environment, were built into the alginate structure, creating additional bonds and thus increasing the scaffold’s strength. The smaller ionic radius of zinc than calcium can facilitate this process, forming a higher chemical bond strength [36]. Secondary cross-linking of the alginate by zinc ions, along with their release, is a possible mechanism. This conclusion was confirmed by the lower zinc concentration in release studies than was expected. Zinc incorporation into the alginate molecular structure was shown via EDS analysis in Figure 6 (Section 3.3). The alginate is composed of irregular blocks of β-d-mannuronic acid (M) and α-l-guluronic acid (G) residues. According to the “egg-box” model, Ca^2+^ binds to GG and MG blocks of alginate, while Zn^2+^ binds to all MM, GG, and MG blocks [26]. Wang et al. described that alginate fibers crosslinked by using Ca-Zn ionic systems demonstrated superior mechanical strength compared with Ca–alginate fibers [37]. The conjugation of two metal ions in alginate fibers during crosslinking had a direct effect on the mechanical strength and swelling properties. The breaking strength and elongation of the Ca–Zn–alginate fibers increased by 40–75%. The authors observed that the bonding stability of metal ions with alginate was higher than that of calcium. The binding mode of the G group in the alginate with zinc ions was double-dentate chelation. The arrangement of M groups was also influenced by the binding force of metal ions.

#### 3.5.3. Structural and Microstructural Changes of Scaffolds After Incubation in PBS (FTIR, SEM/EDS)

**Figure 10:**
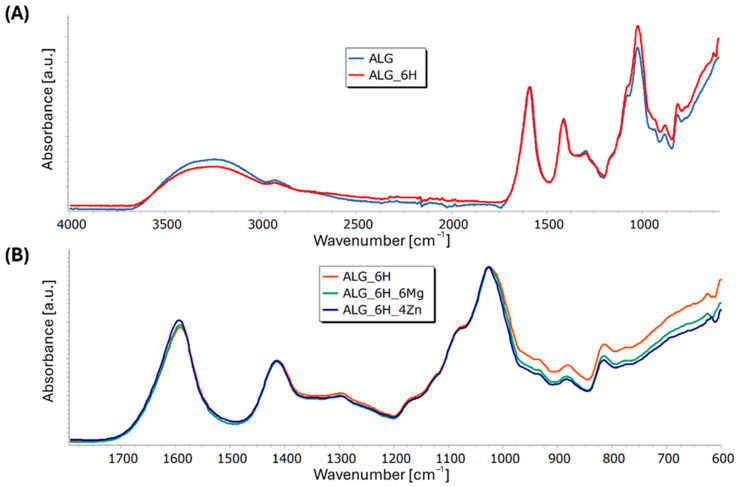
FTIR spectra of ALG_6H scaffold compared to pure alginate (normalized to band at 1593 cm^−1^) (**A**) and composite scaffolds: ALG_6H, ALG_6H_6Mg, and ALG_6H_4Zn (normalized to band at 1025 cm^−1^) (**B**).

The FTIR spectra of the alginate scaffolds showed the bands characteristic of calcium alginate with a max. at 1025 cm^−1^, 1413 cm^−1^, and 1593 cm^−1^, corresponding to the vibrations of the C-O-C and C=O bonds, as well as a max. at 2920 cm^−1^ associated with the vibrations of C-H bonds and approx. 3280 cm^−1^ associated with chemically embedded water (Figure 10A). The clear effect of the addition of HAP on the increase in band intensity can be visible in the range of 920–1200 cm^−1^ for ALG_6H compared to pure alginate (ALG) due to the overlap of alginate bands with bands derived from vibrations in the P-O groups in HAP. Comparing the bands obtained for the ALG_6H, ALG_6H_6Mg, and ALG_6H_4Zn scaffolds (Figure 10B), the effect of the magnesium chloride addition was not visible, while the addition of ZnO caused only slight changes in the intensity of the C=O group band in relation to the other bands and a slight shift of its maximum towards higher wave numbers, as well as a decrease in the half-width of the band with a maximum at 1025 cm^−1^.

**Figure 11:**
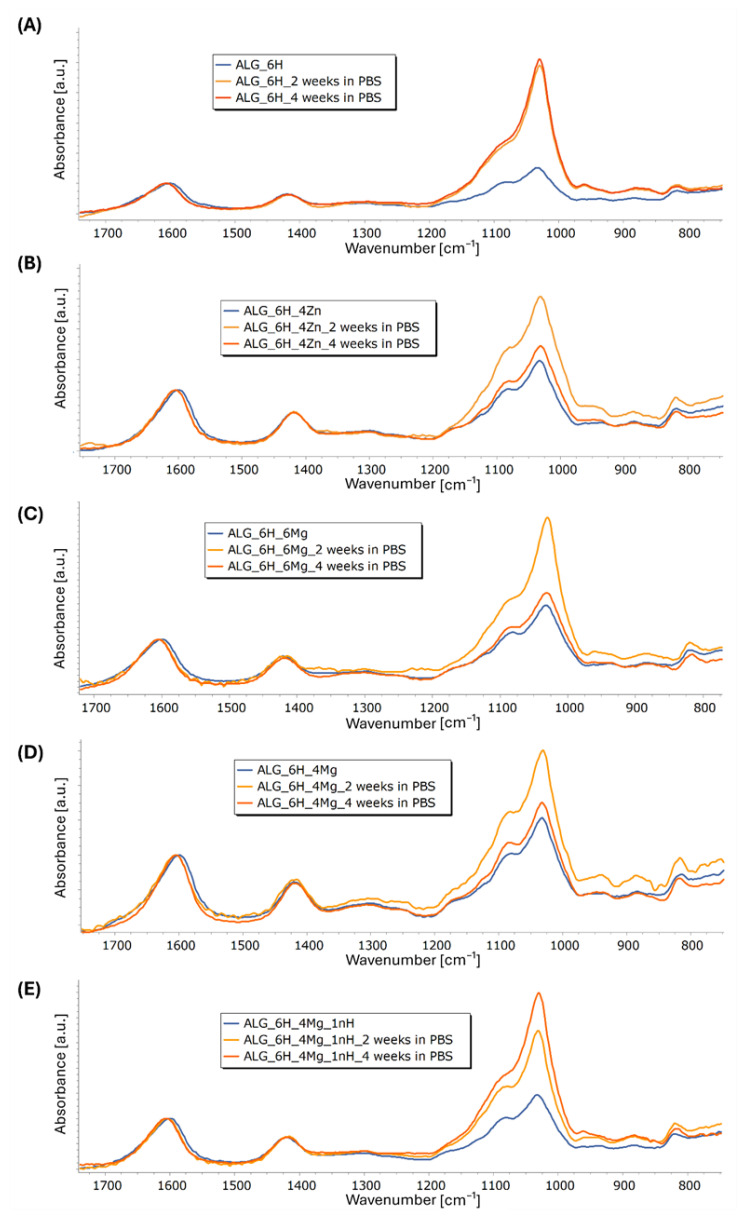
FTIR spectra of alginate scaffolds before and after incubation in PBS: ALG_6H (**A**), ALG_6H_4Zn (**B**), ALG_6H_6Mg (**C**), ALG_6H_4Mg (**D**), and ALG_6H_4Mg_1nH (**E**) (normalized to the band at 1593 cm^−1^).

**Figure 12:**
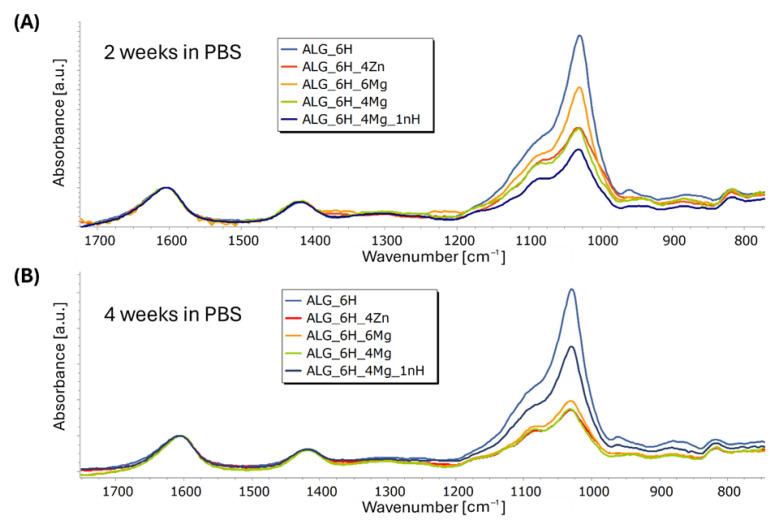
FTIR spectra of alginate scaffolds after 2 (**A**) and 4 (**B**) weeks of incubation in PBS (normalized to the band at 1593 cm^−1^).

Figure 11A–E shows the FTIR spectra obtained for the composite scaffolds comparing initial materials and after 2 and 4 weeks of incubation in PBS. Incubation caused a slight reduction in the half-width of the band with a max. at 1600 cm^−1^ and its slight shift to 1607 cm^−1^ for ALG_6H and to 1605 cm^−1^ for the other scaffolds. This indicates some changes in the molecular structure of the hydrogel during incubation; however, changes indicating alginate degradation were not observed. Moreover, in the case of all materials, a clear increase in the intensity of the bands in the range of 920 to 1200 cm^−1^ in relation to the other bands was observed, associated with an increase in the number of P-O groups. This indicates the formation of apatite secretions during incubation on all tested materials, but the intensity of this process varies. It is clearly visible that in the case of the ALG_6H scaffold (Figure 11A), after 2 weeks, the maximum number of precipitates was reached (the intensity of the bands in the analyzed range is comparable to 4 weeks). Moreover, the highest intensity of these bands for ALG_6H compared to other scaffolds after 2 and 4 weeks of incubation was clearly visible (Figure 12A,B). In the case of ALG_6H_4Zn, ALG_6H_6Mg, and ALG_6H_4Mg (Figure 11B–D), the intensity of these bands (920–1200 cm^−1^) was much higher after 2 than 4 weeks of incubation, which suggests the instability of the originally precipitated apatites. This can be connected with the changes in the environment composition caused by the release of Zn and Mg ions, which altered the nucleation kinetics of the apatites and affected their stability [38,39]. It can be assumed that the new ionic equilibrium has probably stabilized in a closed environment between 2 and 4 weeks of incubation. However, these conditions did not fully reflect the natural biological environment, in which there is a continuous flow of physiological fluids. Teerakanok et al. found that high Zn^2+^ concentration tended to inhibit calcium phosphate crystal formation [38]; however, low-dose Zn ions improve deposition of hydroxyapatite, and Zn was detected in the secretions [40,41]. On the other hand, magnesium promoted the formation and stability of amorphous calcium phosphate, while the transformation of the amorphous phase to hydroxyapatite was inhibited [39]. Yang et al. suggested that Mg^2+^-stabilized amorphous calcium phosphate was a template for hydroxyapatite nucleation [39].

**Figure 13:**
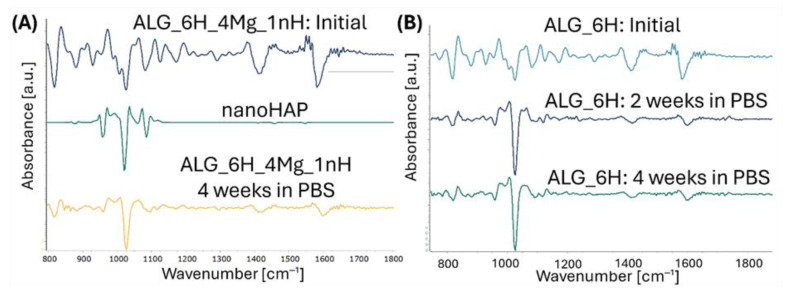
Second derivative of FTIR spectra of scaffolds: ALG_6H_4Mg_1nH initially and after 4 weeks of incubation in PBS compared to nano-HAP (**A**); ALG_6H initially and after 2 and 4 weeks of incubation in PBS (**B**).

Comparing the obtained spectra, a significantly higher intensity of the bands (920–1200 cm^−1^) for ALG_6H_6Mg than for ALG_6H_4Zn and ALG_6H_4Mg was observed, especially after 2 weeks of incubation, which confirms the role of magnesium in the formation of calcium phosphate (Figure 12A,B). EDS analysis of the precipitations for ALG_6H_6Mg showed a disrupted Ca:P ratio compared to the Ca:P ratio in hydroxyapatite (Figure 6A), which confirmed a different creation of calcium phosphates than in hydroxyapatite, in the presence of a high concentration of Mg^2+^. The processes of nucleation and apatite growth were completely different in the case of the ALG_6H_4Mg_1nH scaffold (Figure 11E). The intensity of the bands, depending on the number of P-O groups, increased gradually after 2 and 4 weeks of incubation. Although the analyzed intensity for this sample was lowest after 2 weeks of incubation (Figure 12A), it clearly increased after 4 weeks and was significantly higher compared to the analogous scaffold, but without nano-HAP (ALG_6H_4Mg) and compared to ALG_6H_4Zn and ALG_6H_6Mg. This indicates the significant role of nano-HAP in the formation of stable apatite forms. Because of the overlapping of the bands corresponding with the alginate and HAP in the analyzed range of 920–1200 cm^−1^, the second derivative of the spectra was calculated, which made visible the component bands (Figure 13). It can be visible that the shape of the second derivative after the incubation of the ALG_6H_4Mg_1nH and ALG_6H scaffolds was more similar to nano-HAP than to the initial samples. This confirmed that the bands corresponding with the vibration of P-O groups dominated the bands connected with alginate after incubation because of the presence of a significant amount of apatite secretions. Hydroxyapatite nanoparticles exhibit greater bioactivity due to their large specific surface area and promote the formation of more apatite nucleation centers. Moreover, in the study by Gani et al. [42], bovine-derived HAP nanoparticles (~40 nm) significantly increased osteoblast proliferation, calcium deposition, and expression of osteogenic markers compared to synthetic HAP or HAP microparticles; this also resulted in improved bone growth in an in vivo model. However, hydroxyapatite microparticles, despite their lower bioactivity, are more stable, mechanically reinforce hydrogels, and can be delivered in a higher weight fraction than nano-HAPs, so they can provide a more stable and effective scaffold for bone regeneration in deep osteochondral defects. The simultaneous use of both forms of hydroxyapatite for scaffold modification, in the form of micro and nano particles, gives the opportunity to achieve all benefits at the same time.

**Figure 14:**
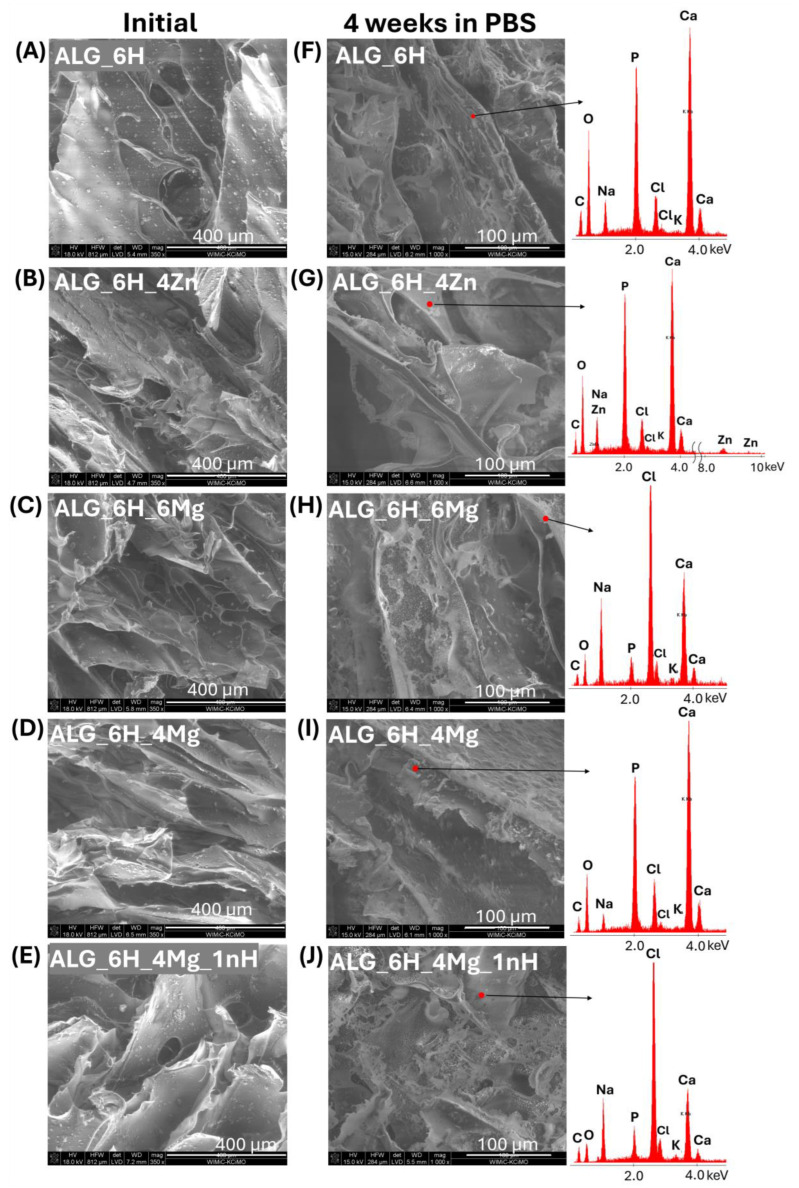
SEM images for initial scaffold (**A**–**E**) and SEM images with EDS analysis in marked points after 4 weeks of incubation (**F**–**J**).

Figure 14A–E shows the SEM microscopic images of the initial alginate scaffolds. A high porosity of the samples could be visible, which facilitated the migration of ions during incubation. A detailed analysis of the microstructure was presented by the authors in a previous paper [43], which showed that porosity was in the range 67–75%. The effect of individual additives on the pore size distribution was also described. Zinc-containing scaffolds showed a modest pore size, indicating a strong pore interconnection. Magnesium-containing scaffolds had significantly larger pore sizes (up to 1430 μm), while the addition of nanohydroxyapatite resulted in the presence of smaller pores (30–310 μm). The observations obtained from FTIR studies were confirmed by SEM/EDS analysis after 4 weeks of incubation in PBS (Figure 14). Microscopic images showed the presence of a significant amount of precipitation on the surface of the scaffolds, which were rich in their content of Ca and P. In the case of scaffolds containing zinc (ALG_6H_4Zn), a significant amount of zinc was incorporated into the created apatites (Figure 14G), as also described in the Section 3.3 with EDS analysis performed after 2 weeks’ incubation in PBS. This indicated that zinc substitutions in apatites occurred after 2 weeks, and these forms were also stable after 4 weeks of incubation. However, magnesium substitutions were not observed in apatites for magnesium chloride-modified samples (Figure 14H,I,J). Magnesium is present in biological apatite [10]; therefore, it was expected that it would also be embedded in apatite precipitation in the performed tests.

## 4. Conclusions

The research showed that the addition of ZnO most effectively improved the mechanical parameters of all types of matrices. Moreover, the part of zinc ions was gradually released into the environment, as well as incorporated into the precipitated apatite. Zinc ions remained in the scaffolds effectively stabilized the alginate structure; therefore, no decrease in their mechanical parameters was observed during the 4 weeks of incubation in PBS. The released zinc ions increased the inhibition zones of *Staphylococcus aureus* growth; however, this effect was observed only for scaffolds with an alginate matrix. For the alginate/gelatin matrix, the influence of zinc was not observed, despite the fact that zinc ions from this matrix were released much faster and in greater quantities. This indicated the key role of the selection of hydrogel for the matrix. Moreover, the alginate matrix showed the best stability during incubation, despite the highest swelling capacity and the lowest initial mechanical parameters.

No effect of magnesium on the inhibition of bacterial growth was observed despite its rapid release. Magnesium ions promoted the efficient secretion of apatite during incubation, which was not stable. The addition of nano-HAP significantly increased the stability of apatite precipitation.

The most promising were scaffolds with an alginate matrix, especially ALG_6H_Zn and ALG_6H_4Mg_1nH, where modifiers had a great impact on their mechanical, physicochemical, and biological properties. These materials could be considered for application as scaffolds for filling and regeneration of osteochondral defects.

In summary, the key advantages of this study were (1) to demonstrate the correlation between the type of hydrogel matrix and the antibacterial activity of ZnO, (2) to uncover the mutual interactions of individual additives in multiphase scaffolds containing micro- and nanometric HAP and magnesium ions on the bioactivity of scaffolds, and (3) the achievement of multifunctionality of scaffolds with HAP and zinc, such as the reinforcement of hydrogels, antibacterial activity, and bioactivity.

## Figures and Tables

**Table 1:** Composition of obtained scaffolds.

No	Code	Composition with wt.%
1	ALG_6H	alginate + 6% HAp
2	ALG_6H_4Mg	alginate + 6% HAp + 1.90% MgCl_2_
3	ALG_6H_6Mg	alginate + 6% HAp + 2.88% MgCl_2_
4	ALG_6H_4Mg_1nH	alginate + 6% HAp + 1.90% MgCl_2_ + 1% nHAp
5	ALG_6H_4Zn	alginate + 6% HAp + 4% ZnO
1	GEL_6H	gelatin + 6% HAp
2	GEL_6H_4Mg	gelatin + 6% HAp + 1.90% MgCl_2_
3	GEL_6H_6Mg	gelatin + 6% HAp + 2.88% MgCl_2_
4	GEL_6H_4Mg_1nH	gelatin + 6% HAp + 1.90% MgCl_2_ + 1% nHAp
5	GEL_6H_4Zn	gelatin + 6% HAp + 4% ZnO
1	ALG/GEL_6H	alginate/gelatin + 6% HAp
2	ALG/GEL_6H_4Mg	alginate/gelatin + 6% HAp + 1.90% MgCl_2_
3	ALG/GEL_6H_6Mg	alginate/gelatin + 6% HAp + 2.88% MgCl_2_
4	ALG/GEL_6H_4Mg_1nH	alginate/gelatin + 6% HAp + 1.90% MgCl_2_ + 1% nHAp
5	ALG/GEL_6H_4Zn	alginate/gelatin + 6% HAp + 4% ZnO

**Table 2:** Inhibition zones of *Staphylococcus aureus* growth after 24 h of incubation (the distance between the edge of the sample and the distinct edge of the bacterial growth zone was measured).

Matrix	Inhibition Zone [mm]
ALG_6H	3.2 ± 0.3
ALG_6H_6Mg	3.0 ± 1.7
ALG_6H_4Zn	5.0 ± 0
ALG/GEL_6H	1.0 ± 0
ALG/GEL_6H_6Mg	0.6 ± 0.3
ALG/GEL_6H_4Zn	1.0 ± 0

## Data Availability

The original contributions presented in the study are included in the article, further inquiries can be directed to the corresponding author.

## References

[1] Tsujii A., Ohori T., Hanai H., Nakamura N. (2024). Next Generation Approaches for Cartilage Repair and Joint Preservation. J. Cartil. Jt. Preserv..

[2] Cong B., Sun T., Zhao Y., Chen M. (2023). Current and Novel Therapeutics for Articular Cartilage Repair and Regeneration. Ther. Clin. Risk Manag..

[3] Haung S.-M., Lin Y.-T., Liu S.-M., Chen J.-C., Chen W.-C. (2021). In Vitro Evaluation of a Composite Gelatin–Hyaluronic Acid–Alginate Porous Scaffold with Different Pore Distributions for Cartilage Regeneration. Gels.

[4] Sharma S., Bhende M., Goel A. (2024). A Review: Polysaccharide-Based Hydrogels and Their Biomedical Applications. Polym. Bull..

[5] Tomić S.L., Nikodinović-Runić J., Vukomanović M., Babić M.M., Vuković J.S. (2021). Novel Hydrogel Scaffolds Based on Alginate, Gelatin, 2-Hydroxyethyl Methacrylate, and Hydroxyapatite. Polymers.

[6] Ramesh N., Moratti S.C., Dias G.J. (2018). Hydroxyapatite-Polymer Biocomposites for Bone Regeneration: A Review of Current Trends. J. Biomed. Mater. Res. B Appl. Biomater..

[7] Kavitha Sri A., Arthi C., Neya N.R., Hikku G.S. (2023). Nano-Hydroxyapatite/Collagen Composite as Scaffold Material for Bone Regeneration. Biomed. Mater..

[8] Zhao W., Sun B., Song Y., Cao Y., Liu Y., Zhou D., Zhou Q., Xie F., Huang W., Li X. (2025). Nanohydroxyapatite and Liposomes-Coated Integral Bilayer Scaffold for Osteochondral Repair via Mimicking the Dual Differentiation Microenvironment of BMSCs. Nano Mater. Sci..

[9] Zhou H., Liang B., Jiang H., Deng Z., Yu K. (2021). Magnesium-Based Biomaterials as Emerging Agents for Bone Repair and Regeneration: From Mechanism to Application. J. Magnes. Alloys.

[10] Fatima G., Dzupina A., Alhmadi H.B., Magomedova A., Siddiqui Z., Mehdi A., Hadi N. (2024). Magnesium Matters: A Comprehensive Review of Its Vital Role in Health and Diseases. Cureus.

[11] Yuan Z., Lyu Z., Liu X., Zhang J., Wang Y., Yuan Z., Lyu Z., Liu X., Zhang J., Wang Y. (2021). Mg-BGNs/DCECM Composite Scaffold for Cartilage Regeneration: A Preliminary In Vitro Study. Pharmaceutics.

[12] Zhao J., Wu H., Wang L., Jiang D., Wang W., Yuan G., Pei J., Jia W. (2022). The Beneficial Potential of Magnesium-Based Scaffolds to Promote Chondrogenesis through Controlled Mg2+ Release in Eliminating the Destructive Effect of Activated Macrophages on Chondrocytes. Biomater. Adv..

[13] Xie K., Wang N., Guo Y., Zhao S., Tan J., Wang L., Li G., Wu J., Yang Y., Xu W. (2022). Additively Manufactured Biodegradable Porous Magnesium Implants for Elimination of Implant-Related Infections: An in Vitro and in Vivo Study. Bioact. Mater..

[14] Alarcón P.O., Sossa K., Contreras D., Urrutia H., Nocker A. (2014). Antimicrobial Properties of Magnesium Chloride at Low PH in the Presence of Anionic Bases. Magnes. Res..

[15] Mousa H.M., Abdal-Hay A., Bartnikowski M., Mohamed I.M.A., Yasin A.S., Ivanovski S., Park C.H., Kim C.S. (2018). A Multifunctional Zinc Oxide/Poly(Lactic Acid) Nanocomposite Layer Coated on Magnesium Alloys for Controlled Degradation and Antibacterial Function. ACS Biomater. Sci. Eng..

[16] Shitole A.A., Raut P.W., Sharma N., Giram P., Khandwekar A.P., Garnaik B. (2019). Electrospun Polycaprolactone/Hydroxyapatite/ZnO Nanofibers as Potential Biomaterials for Bone Tissue Regeneration. J. Mater. Sci. Mater. Med..

[17] Nair S., Sasidharan A., Divya Rani V.V., Menon D., Nair S., Manzoor K., Raina S. (2009). Role of Size Scale of ZnO Nanoparticles and Microparticles on Toxicity toward Bacteria and Osteoblast Cancer Cells. J. Mater. Sci. Mater. Med..

[18] Bashir S., Awan M.S., Farrukh M.A., Naidu R., Khan S.A., Rafique N., Ali S., Hayat I., Hussain I., Khan M.Z. (2022). In-Vivo (Albino Mice) and in-Vitro Assimilation and Toxicity of Zinc Oxide Nanoparticles in Food Materials. Int. J. Nanomed..

[19] Kwiecień K., Knap K., Heida R., Czajkowski J., Gorter A., Ochońska D., Mielczarek P., Dorosz A., Niewolik D., Reczyńska-Kolman K. (2025). Novel Copolymers of Poly(Sebacic Anhydride) and Poly(Ethylene Glycol) as Azithromycin Carriers to the Lungs. Biocybern. Biomed. Eng..

[20] Li Z., Liu H., Liao Y., Wang H., Sun X., Chen X., Yan H., Lin Q. (2023). Design and Properties of Alginate/Gelatin/Cellulose Nanocrystals Interpenetrating Polymer Network Composite Hydrogels Based on in Situ Cross-Linking. Eur. Polym. J..

[21] Ko C.L., Wu H.Y., Lin Y.S., Yang C.H., Chen J.C., Chen W.C. (2017). Modulating the Release of Proteins from Aloaded Carrier of Alginate/Gelatin Porous Spheres Immersed in Different Solutions. Biomed. Mater. Eng..

[22] Vedadghavami A., Minooei F., Mohammadi M.H., Khetani S., Rezaei Kolahchi A., Mashayekhan S., Sanati-Nezhad A. (2017). Manufacturing of Hydrogel Biomaterials with Controlled Mechanical Properties for Tissue Engineering Applications. Acta Biomater..

[23] Tan J., Luo Y., Guo Y., Zhou Y., Liao X., Li D., Lai X., Liu Y. (2023). Development of Alginate-Based Hydrogels: Crosslinking Strategies and Biomedical Applications. Int. J. Biol. Macromol..

[24] Gong H., Zi Y., Kan G., Li L., Shi C., Wang X., Zhong J. (2024). Preparation of Food-Grade EDC/NHS-Crosslinked Gelatin Nanoparticles and Their Application for Pickering Emulsion Stabilization. Food Chem..

[25] Chen P.R., Kang P.L., Su W.Y., Lin F.H., Chen M.H. (2005). The Evaluation of Thermal Properties and in Vitro Test of Carbodiimide or Glutaraldehyde Cross-Linked Gelatin for PC 12 Cells Culture. Biomed. Eng.-Appl. Basis Commun..

[26] Malektaj H., Drozdov A.D., de Claville Christiansen J. (2023). Mechanical Properties of Alginate Hydrogels Cross-Linked with Multivalent Cations. Polymers.

[27] Innocentini M.D.D.M., Rasteira V.D., Potoczek M., Chmielarz A., Kocyło E. (2017). Physical, Fluid Dynamic and Mechanical Properties of Alumina Gel-Cast Foams Manufactured Using Agarose or Ovalbumin as Gelling Agents. J. Mater. Res..

[28] Shaheen A., Maswal M., Dar A.A. (2021). Synergistic Effect of Various Metal Ions on the Mechanical, Thixotropic, Self-Healing, Swelling and Water Retention Properties of Bimetallic Hydrogels of Alginate. Colloids Surf. A Physicochem. Eng. Asp..

[29] Peppas N.A., Bures P., Leobandung W., Ichikawa H. (2000). Hydrogels in Pharmaceutical Formulations. Eur. J. Pharm. Biopharm..

[30] Luque-Agudo V., Fernández-Calderón M.C., Pacha-Olivenza M.A., Pérez-Giraldo C., Gallardo-Moreno A.M., González-Martín M.L. (2020). The Role of Magnesium in Biomaterials Related Infections. Colloids Surf. B Biointerfaces.

[31] Kang S.N., Jeong C.M., Jeon Y.C., Byon E.S., Jeong Y.S., Cho L.R. (2014). Effects of Mg-Ion and Ca-Ion Implantations on P. Gingivalis and F. Nucleatum Adhesion. Tissue Eng. Regen. Med..

[32] De Kerchove A.J., Elimelech M. (2008). Calcium and Magnesium Cations Enhance the Adhesion of Motile and Nonmotile Pseudomonas Aeruginosa on Alginate Films. Langmuir.

[33] Meißner T., Oelschlägel K., Potthoff A. (2014). Implications of the Stability Behavior of Zinc Oxide Nanoparticles for Toxicological Studies. Int. Nano Lett..

[34] Yuan Z., Wei P., Huang Y., Zhang W., Chen F., Zhang X., Mao J., Chen D., Cai Q., Yang X. (2019). Injectable PLGA Microspheres with Tunable Magnesium Ion Release for Promoting Bone Regeneration. Acta Biomater..

[35] Valadbeigi H., Sadeghifard N., Kaviar V.H., Haddadi M.H., Ghafourian S., Maleki A. (2023). Effect of ZnO Nanoparticles on Biofilm Formation and Gene Expression of the Toxin-Antitoxin System in Clinical Isolates of Pseudomonas Aeruginosa. Ann. Clin. Microbiol. Antimicrob..

[36] Xu P., Wang H., Ren L., Tu B., Wang W., Fu Z. (2021). Theoretical Study on Composition-Dependent Properties of ZnO·nAl_2_O_3_ Spinels. Part II: Mechanical and Thermophysical. J. Am. Ceram. Soc..

[37] Wang Q., Zhang L., Liu Y., Zhang G., Zhu P. (2020). Characterization and Functional Assessment of Alginate Fibers Prepared by Metal-Calcium Ion Complex Coagulation Bath. Carbohydr. Polym..

[38] Teerakanok S., Zhao M., Giordano R., Fan Y. (2021). Interaction of Doped Magnesium, Zinc and Fluoride Ions on Hydroxyapatite Crystals Grown on Etched Human Enamel. J. Cryst. Growth.

[39] Yang X., Xie B., Wang L., Qin Y., Henneman Z.J., Nancollas G.H. (2011). Influence of Magnesium Ions and Amino Acids on the Nucleation and Growth of Hydroxyapatite. CrystEngComm.

[40] Alessandri Bonetti G., Pazzi E., Zanarini M., Marchionni S., Checchi L. (2014). The Effect of Zinc-Carbonate Hydroxyapatite versus Fluoride on Enamel Surfaces after Interproximal Reduction. Scanning.

[41] Lelli M., Putignano A., Marchetti M., Foltran I., Mangani F., Procaccini M., Roveri N., Orsini G. (2014). Remineralisation and Repair of Enamel Surface by Biomimetic Zn-Carbonate Hydroxyapatite Containing Toothpaste: A Comparative in Vivo Study. Front. Physiol..

[42] Gani M.A., Lee G., Ardianto C., Rantam F.A., Lestari M.L.A.D., Addimaysqi R., Ketut Adnyana I., Lee K., Khotib J. (2025). Comparative Study of Bovine and Synthetic Hydroxyapatite in Micro- and Nanosized on Osteoblasts Action and Bone Growth. PLoS ONE.

[43] de Mello Innocentini M.D., Fuzatto Bueno B.R., Urbaś A., Morawska-Chochół A. (2024). Microstructural, Fluid Dynamic, and Mechanical Characterization of Zinc Oxide and Magnesium Chloride-Modified Hydrogel Scaffolds. ACS Biomater. Sci. Eng..

